# The case for caution in the application of whole-exome sequencing data for immune repertoire analysis

**DOI:** 10.1093/bib/bbag419

**Published:** 2026-08-05

**Authors:** Zheng Gong, Weixin Zhang, Bingyan Du, Hongkai Wu, Shiyue Li

**Affiliations:** State Key Laboratory of Respiratory Disease, National Clinical Research Center for Respiratory Disease, The First Affiliated Hospital of Guangzhou Medical University, Guangzhou Medical University, 195 Dongfengxi Road, Yuexiu District, Guangzhou, Guangdong Province, 510182, China; State Key Laboratory of Respiratory Disease, National Clinical Research Center for Respiratory Disease, The First Affiliated Hospital of Guangzhou Medical University, Guangzhou Medical University, 195 Dongfengxi Road, Yuexiu District, Guangzhou, Guangdong Province, 510182, China; State Key Laboratory of Respiratory Disease, National Clinical Research Center for Respiratory Disease, The First Affiliated Hospital of Guangzhou Medical University, Guangzhou Medical University, 195 Dongfengxi Road, Yuexiu District, Guangzhou, Guangdong Province, 510182, China; State Key Laboratory of Respiratory Disease, National Clinical Research Center for Respiratory Disease, The First Affiliated Hospital of Guangzhou Medical University, Guangzhou Medical University, 195 Dongfengxi Road, Yuexiu District, Guangzhou, Guangdong Province, 510182, China; State Key Laboratory of Respiratory Disease, National Clinical Research Center for Respiratory Disease, The First Affiliated Hospital of Guangzhou Medical University, Guangzhou Medical University, 195 Dongfengxi Road, Yuexiu District, Guangzhou, Guangdong Province, 510182, China

**Keywords:** adaptive immune receptor repertoire, immune repertoire sequencing, whole-exome sequencing, RNA sequencing, T-cell receptor, B-cell receptor

## Abstract

The analysis of the adaptive immune receptor repertoire is critical in disease research. While specialized immune repertoire sequencing (IR-seq) is the dedicated method for receptor repertoire profiling, the abundance of existing WES data has led to its frequent repurposing for repertoire analysis; however, its reliability for this application remains poorly evaluated. To address this, we first assessed the VDJ gene coverage by commercial WES probes. Next, using a multi-omics dataset (matched IR-seq, RNA-seq, WES), we compared repertoire inferences derived from WES and RNA-seq results against IR-seq. This comparison was followed by an analysis correlating WES probe matching efficiency with the failure to identify V/J gene segments. We found that WES probes miss a substantial fraction of individual V, D, and J genes and provide uneven coverage across these genes. WES consistently showed significantly lower performance than RNA-seq, detecting fewer clonotypes and yielding greater abundance deviation. The underestimation of clonotype richness in WES was confirmed through external validation using multiple public datasets. Our results illustrate the inherent limitations of WES for repertoire profiling, serving as a cautionary note for the appropriate interpretation of exome-derived repertoire information in research.

## Introduction

The adaptive immune system is governed by antigen-specific recognition mediated by T-cell receptors (TCRs) and B-cell receptors (BCRs), collectively comprising the adaptive immune receptor repertoire (AIRR). Generated through VDJ recombination, the AIRR attains immense molecular diversity that underlies immune surveillance and host defense [1]. Changes in AIRR composition and diversity serve as sensitive biomarkers of immune activity in cancer, infection, and autoimmunity, and repertoire dynamics have been linked to disease progression and therapeutic response [2–4]. Consequently, systematic profiling of the AIRR both deepens mechanistic understanding and informs precision-medicine strategies.

The AIRR research is fundamentally based on high-throughput sequencing data [5, 6]. In specialized studies, the sequencing libraries that capture the hypervariable regions of the TCR and BCR chains are constructed via immune repertoire sequencing (IR-seq). This method typically employs either multiple primers complementary to V and J gene families, or the 5′ rapid amplification of cDNA ends (5′ RACE) approach, which uses a primer in the constant region of the cDNA [7]. In contrast, data generated by more widely available methods, such as RNA sequencing (RNA-seq) and whole-exome sequencing (WES), are abundant in clinical settings and public repositories. These datasets are frequently utilized for AIRR analysis. Although most sequencing reads are not derived from TCR or BCR regions, even in samples enriched for lymphocytes, repurposing existing WES or RNA-seq data for repertoire profiling may be cost-effective. While RNA-seq theoretically sequences transcribed DNA without major bias toward dominant clonotypes and is considered effective at capturing clonotypes and estimating TCR repertoire diversity [8, 9], WES relies on a designed set of probes to capture DNA from specific exonic regions of the genome [10]. Although WES data have been reported for AIRR research in ~50 studies over the past 10 years (Supplementary Table S1), most of these studies represent repurposing of existing datasets, and the methodological reliability of WES for AIRR analysis remains to be thoroughly evaluated.

In this study, we first evaluated the correspondence between the V, D, and J gene segments and several commonly used commercial WES probes, considering both genomic coordinates and sequence similarity. Then, using a multi-omics dataset containing WES, RNA-seq, and IR-seq data, we compared the WES and RNA-seq results with the AIRR clonotype identification results from IR-seq serving as the gold standard. This comparison was followed by an analysis exploring the relationship between probe matching efficiency to the V/J gene segments and the failure to identify V/J gene segments in WES. Finally, we validated the comparative results using several publicly available datasets containing paired WES and RNA-seq data. Our results illustrated the capabilities and limitations of WES for AIRR profiling, offering insights into the appropriate and cautious use of exome-derived repertoire information.

## Results

### VDJ gene capture by whole-exome sequencing probes is inherently nonuniform

Given the critical role of WES probes in capturing the complete V, D, and J gene repertoire, our initial step was to determine the probes’ genomic coordinates and their sequence matching efficiency against the VDJ segments. In this analysis, all seven chains of TCR and BCR (TRA, TRB, TRD, TRG, IGH, IGK, and IGL) were considered.

**Figure 1:**
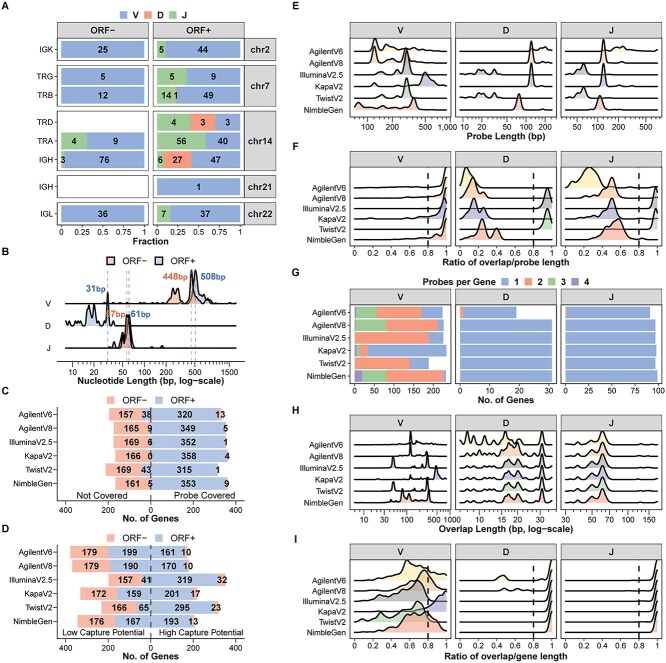
Positional features of VDJ genes and computational assessment of exome probe capture across commercial platforms. (A) Chromosomal distribution of VDJ genes (*n* = 528), stratified by gene type (V, D, J) and functional status (ORF+/ORF−). (B) Nucleotide length distribution of VDJ genes (*n* = 528), stratified by functional status (ORF− and ORF+), as indicated in the figure legend. Dashed lines indicate the length peaks for ORF− and ORF+ genes within each gene type. (C) Number of VDJ genes (*n* = 528) classified according to genomic coverage by probes (probe covered or not covered) across six WES platforms, stratified by functional status. (D) Number of VDJ reference genes (*n* = 549) classified into high and low capture potential categories based on BLASTn alignment of probe sequences against VDJ reference gene sequences, stratified by functional status. (E) Nucleotide length distribution of probes overlapping VDJ genes’ genomic regions across six platforms. (F) Ratio distribution of probe–gene overlap length to probe length across six platforms, stratified by gene type. Dashed lines indicate a ratio of 0.8. (G) Number of probes per covered gene across six platforms, stratified by gene type. (H) Distribution of total probe–gene overlap lengths per gene across six platforms, stratified by gene type. (I) Ratio distribution of total probe–gene overlap length to gene length across six platforms, stratified by gene type. Dashed lines indicate a ratio of 0.8. ORF−: genes classified as nonfunctional based on their annotation source; ORF+: genes classified as functional. See footnotes in Supplementary Tables S3 (A–C) and 4 (D) for detailed definitions. TRA, T-cell receptor alpha chain; TRB, T-cell receptor beta chain; TRD, T-cell receptor delta chain; TRG, T-cell receptor gamma chain; IGH, immunoglobulin heavy chain; IGL, immunoglobulin light-chain lambda; IGK, immunoglobulin light-chain kappa.

The names of 549 VDJ genes were obtained from the MiXCR internal library. Of these, 528 genes were recorded in Ensembl (GRCh38 v113), 170 of which lack open reading frames (ORF−) (Fig. 1A). In terms of genomic gene length, the D (~20 bp) and J (~50 bp) genes are relatively short, while V genes are much longer (~500 bp) (Fig. 1B). We compared the genomic coordinates of six commercial probe sets (Supplementary Table S2) against the positions of 528 VDJ genes retrieved from Ensembl (Supplementary Table S3; Supplementary Figs S1–S4). Here, a gene was considered covered if its genomic coordinate region overlapped with those of any of the probes. For six platforms, only ~65% of the VDJ genes were covered by the commercial probes, and most of these covered genes contained intact open reading frames (ORF+) (Fig. 1C). The probes that covered the VDJ genes exhibited a distribution in length, and different manufacturers employed the varying probe length strategies (Fig. 1E). We then calculated the ratio of gene–probe overlap length to probe length. For the D and J genes, only two platforms (IlluminaV2.5 and TwistV2) demonstrated a ratio near 1.0, while the remaining platforms exhibited comparatively low ratios (Fig. 1F). For the V genes, the ratio distribution not only peaked at 1.0 but also showed a low-ratio tail, indicating incomplete coverage for a subset of V gene probes (Fig. 1F). Importantly, the number and overall genomic coverage of probes differed significantly among the targeted genes. Typically, only a single probe was designed per D and J gene. In contrast, the V genes were often covered by multiple probes (two to four probes) per gene (Fig. 1G). For each gene, we quantified the total overlap length between the gene and probes. The results demonstrated a heterogeneous distribution of overlap lengths across the gene set (Fig. 1H). To evaluate the actual completeness of gene coverage regardless of probe length and tiling strategies, we further quantified the ratio of total probe–gene overlap length to the respective gene length (Fig. 1I). The results revealed considerable heterogeneity in coverage completeness across individual genes within the same gene type. For V genes, the ratio distribution was broad, indicating that while some genes achieved near-complete coverage, a substantial proportion remained only partially covered even with multi-probe tiling strategies. For D and J genes, while the ratio distribution was largely concentrated at 1.0, some intergene variation in coverage completeness was nevertheless observed. To further assess coverage at the biologically relevant ends of V and J genes, we aligned all V genes to their 3′ end and all J genes to their 5′ end and calculated the percentage of genes with probe coverage at each relative position (Supplementary Fig. S5). For V genes, probe coverage increased progressively toward the recombination-proximal 3′ end, yet even at this most critical position, coverage did not reach 100% across any platform (Supplementary Fig. S5A). This further supports the conclusion that WES probe sets provide incomplete and uneven coverage of V genes, even at the regions of greatest biological relevance for detecting rearranged receptors. For J genes, probe coverage was generally high across the full gene length, though coverage at the recombination-proximal 5′ end did not reach 100% on all platforms (Supplementary Fig. S5B).

Since gene capture by probes fundamentally relies on sequence complementarity, we adopted an alignment-based strategy to investigate the similarity between the probes and VDJ genes. To ensure the analysis was consistent with the general VDJ gene identification process, we collected 549 human VDJ gene sequences and their associated functional status (ORF+/ORF-) from the built-in MiXCR reference libraries (^*^00 allele). In MiXCR, the ^*^00 allele is a consensus sequence derived from all known alleles of a given gene, serving as a gene-level representative rather than any specific allelic variant. Subsequently, all commercial probe sequences (used as queries derived from genomic coordinates, irrespective of their overlap with the VDJ gene regions) were aligned against these VDJ gene reference sequences using BLASTn. In the BLASTn results, the probe corresponding to the high scoring pair (HSP) with the highest bit score was considered the most likely to hybridize with the gene. Therefore, the HSP similarity parameters could be used to estimate the gene’s capture efficiency by the probe. For simplicity, we established thresholds of pident (percent identity) ≥ 90% and qcovhsp (query coverage per HSP) ≥ 80% to categorize genes into two groups: those with high versus low potential to be captured, where a gene was classified as having high capture potential if it yielded the best-scoring HSP meeting both criteria (Fig. 1D; Supplementary Table S4). The results indicate that, except for the IlluminaV2.5 and TwistV2 platforms, approximately two-thirds of the VDJ genes for the other four platforms fell into the low capture potential classification. It is worth mentioning that many ORF− genes were classified into the high capture category. This is attributed to the fact that these nonfunctional genes often originate from mutations within gene families that maintain high sequence similarity to their ORF+ members [11].

In summary, by design, WES probes are expected to miss a substantial fraction of individual V, D, and J genes and provide uneven coverage across these genes. Furthermore, the analysis of sequence similarity suggests that the likelihood of different VDJ genes being captured by the probes is also variable.

### WES performance was inferior to RNA-seq in receptor clonotype profiling: analysis of a three-method sequencing dataset

We used the dataset derived from multiple human liver tissue samples, including 64 tumor and 64 adjacent nontumor tissues from hepatocellular carcinoma (HCC) patients [12]. For each sample, there were Illumina paired-end sequencing results from WES (using the Agilent V6 platform), RNA-seq, and IR-seq. The sequencing depths were 65.55 ± 16.19 million reads for WES (150 bp read length), 48.47 ± 6.08 million reads for RNA-seq (150 bp read length), and 5.24 ± 0.73 million reads for IR-seq (250 bp read length). The IR-seq method targeted the TRA, TRB, TRD, TRG, and IGH loci. BCR light chains (IGK and IGL) were not targeted in the IR-seq protocol and therefore are not included in this analysis. The dataset was utilized to identify and quantify TCR/BCR clonotypes. The results obtained from WES and RNA-seq were then compared against the IR-seq results, which served as the gold standard.

**Figure 2:**
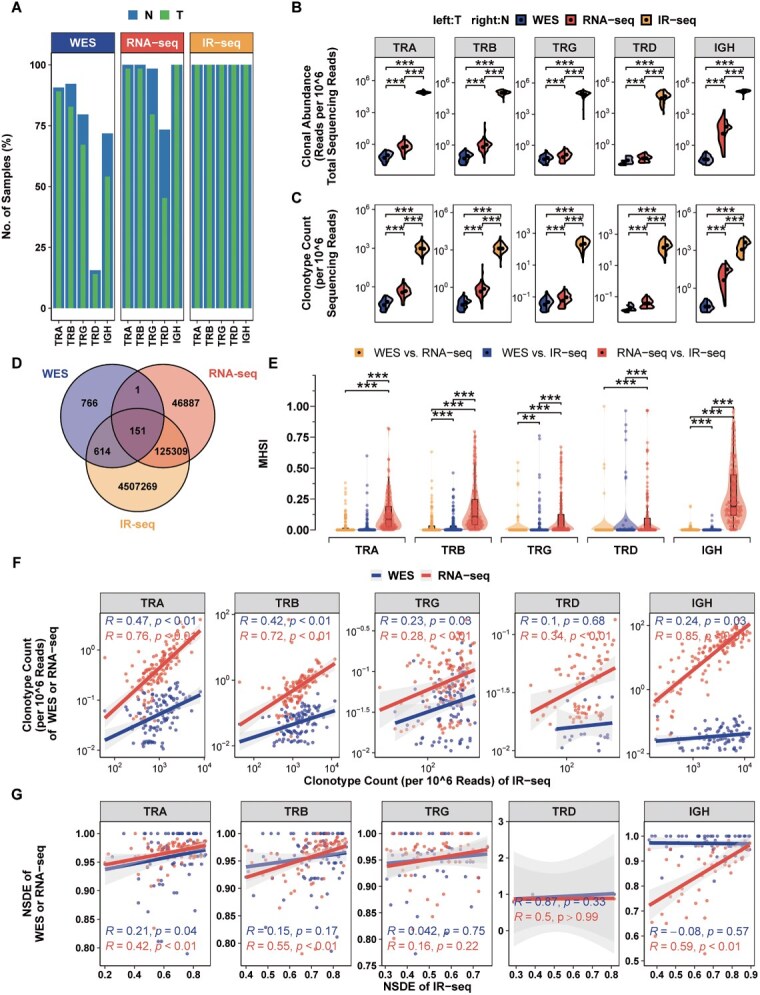
Performance of WES in clonotype profiling compared with RNA-seq and IR-seq. (A) Percentage of samples with detectable TCR/BCR clonotypes across three sequencing methods (WES, RNA-seq, and IR-seq), stratified by sample type (T, tumor; N, adjacent nontumor). (B) Violin plots of clonal abundance (number of reads belonging to clonotypes per 10^6^ sequencing reads) across three sequencing methods. The left-hand violin denotes tumor samples (T), and the right-hand violin denotes adjacent nontumor samples (N); central points indicate means. (C) Violin plots of clonotype count (number of identified clonotypes per 10^6^ sequencing reads) across three sequencing methods. The visualization style follows that described in (B). (D) Venn diagram of clonotypes shared among WES, RNA-seq, and IR-seq. (E) MHSI of clonotype abundance profiles between pairwise methods. (F) Spearman correlation of clonotype counts. Each point represents paired clonotype counts for a single sample, with IR-seq values on the x-axis and WES or RNA-seq values on the y-axis, as identified in the figure legend. Solid lines are regression fits and shaded bands are 95% confidence intervals. The upper and lower sets of statistical annotations in each panel report the correlation coefficient (*R*) and *p*-value for WES versus IR-seq and RNA-seq versus IR-seq, respectively. (G) Spearman correlation of NSDE. The visualization style follows that described in (F). Analyses were performed on 64 paired tumor (T) and adjacent nontumor (N) samples (128 samples in total) from the HCC dataset, each with matched WES, RNA-seq, and IR-seq data. For IGH analyses, three samples were excluded owing to nucleic acid extraction failure during IR-seq IGH library preparation (*n* = 125 for IGH; *n* = 128 for TCR chains). TRA, T-cell receptor alpha chain; TRB, T-cell receptor beta chain; TRD, T-cell receptor delta chain; TRG, T-cell receptor gamma chain; IGH, immunoglobulin heavy chain; MHSI, Morisita–Horn similarity index; NSDE, normalized Shannon diversity entropy. Statistical comparisons: Wilcoxon signed-rank test; ^*^P < .05, ^**^P < .01, ^***^P < .001.

We first assessed the detectability of receptor sequence clonotypes in each sequencing sample. IR-seq identified clonotypes for all five chains in all samples. RNA-seq detection failed in 1% of samples for TRA and TRB chains, and in 11% (TRD) and 41% (TRG) of samples. WES exhibited the lowest detection rates across all chains, notably identifying TRD clonotypes in just 15% of samples (Fig. 2A). For samples in which clonotypes were identified, we quantified clonal abundance by counting the number of reads belonging to receptor clones and normalizing this count according to the sample’s sequencing depth. The results showed that, as expected, clonal sequences were most abundant in IR-seq data, while their abundance in WES data was lower than that in RNA-seq (Fig. 2B; Supplementary Table S5). In terms of clonotype count, the difference in detection capability across the methods was marked. For example, for the TRB chain, IR-seq detected a median of 6873 clonotypes (interquartile range [IQR]: 4286 - 11,941), RNA-seq detected 31 clonotypes (IQR: 14.5–53.5), while WES detected only 4 clonotypes (IQR: 2–6). Across all five chains, when normalized to per million sequencing reads, IR-seq consistently yielded the highest number of clonotypes, followed by RNA-seq, with WES detecting the lowest number (Fig. 2C; Supplementary Table S5).

We examined the consistency of clonotype identification results across the three methods. Overall, ~73% of clonotypes identified in RNA-seq were also identified in IR-seq, compared to only ~50% in WES (Fig. 2D). The abundance of each clonotype was calculated for every method. We then utilized the Morisita–Horn similarity index (MHSI) to assess the consistency of the clonotype abundance profiles between pairwise methods for each sample’s repertoire. The MHSI value ranges from 0 (complete inconsistency) to 1 (complete consistency). If a clonotype was not detected by one of the methods in a comparison, its abundance in that method was considered to be 0. The results showed that the repertoire clonotype abundance determined by RNA-seq was markedly more consistent with IR-seq than that determined by WES. Furthermore, the consistency observed between WES and RNA-seq was also poor (Fig. 2E; Supplementary Table S5).

As AIRR analysis often requires assessing sample richness and evenness, we examined the correlation of these metrics between the methods. Specifically, we correlated richness (measured by the number of clonotypes per million sequencing reads) and evenness (measured by the normalized Shannon diversity entropy, NSDE) for the RNA-seq versus IR-seq and WES versus IR-seq pairwise comparisons. We found that for richness, the RNA-seq/IR-seq correlation was significantly higher than the WES/IR-seq correlation across all five chains (Fig. 2F). Regarding evenness, the RNA-seq/IR-seq correlation also surpassed the WES/IR-seq correlation for all chains except TRD (Fig. 2G).

In summary, based on the evaluation results derived from this three-method dataset (WES, RNA-seq, and IR-seq), WES consistently demonstrated lower performance compared to RNA-seq. Specifically, WES detected fewer clonal sequences and clonotypes in the samples. Meanwhile, the clonotype abundance quantified by WES showed a greater deviation from the IR-seq gold standard than that quantified by RNA-seq.

### Evidence for probe match efficiency limiting clonotype detection in whole-exome sequencing

Given the significantly lower number of clonotypes detected by WES compared to the other methods, we further investigated the underlying basis by examining the relationship between the probe’s gene capture ability and the observed low detection rate.

**Figure 3:**
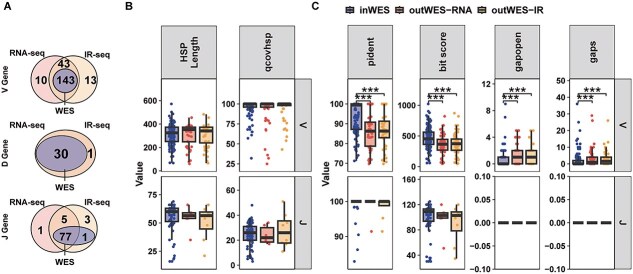
Relationship between the detectability of V and J genes in WES and probe–gene sequence matching metrics. (A) Overlap of V, D, and J genes detected by WES, RNA-seq, and IR-seq in the same HCC dataset as Fig. 2. Based on the clonotype identification results, a VDJ gene was considered detected by a sequencing method if it appeared in at least one clonotype identified from any sample analyzed by that method. (B) HSP length and qcovhsp for V and J genes, grouped by their detection status across the three sequencing methods. Genes were divided into three groups: inWES, genes detected by WES; outWES-RNA, genes not detected by WES but detected by RNA-seq; and outWES-IR, genes not detected by WES but detected by IR-seq. (C) HSP similarity metrics of V and J genes across the same three groups. Metrics include pident, bit score, gapopen, and gaps. Each point in (B) and (C) represents one gene. HSP metrics were derived from BLASTn alignment of WES probe sequences against VDJ gene reference sequences, using the best-scoring HSP for each gene. HSP, high scoring pair; qcovhsp, query coverage per HSP, calculated as the percentage of probe length covered by the HSP; pident, percentage of identical bases; bit score, similarity score generated by BLASTn; gapopen, number of gap openings in the HSP; gaps, total number of bases within all gaps in the HSP. Statistical comparisons: Wilcoxon rank-sum test; ^*^P < .05, ^**^P < .01, ^***^P < .001.

We began by examining the occurrence of VDJ genes across the analyzed loci (TRA, TRB, TRD, TRG, and IGH) in the HCC dataset, based on the clonotype identification results described above. A VDJ gene was considered present in a method if it was identified as the best-hit match for at least one clonotype in any sample analyzed by that method. The results showed that a total of 209 V genes, 31 D genes, and 87 J genes were detected across the three sequencing methods. For all three gene segments, the genes detected by the WES method were consistently subsets of those detected by RNA-seq and IR-seq methods. Specifically, WES detected 143 V genes (68%), 30 D genes (97%), and 78 J genes (90%) of the total gene pool (Fig. 3A). The high detection rate of the D genes (97%) may be attributed to their central position flanked by the V and J segments within rearranged loci [13]. Detection of a portion of D genes likely occurs not through their own specific probe, but rather through co-capture by the probes targeting the adjacent V or J gene segments [14].

We further focused on the V and J genes to assess their sequence similarity to probes based on the HSP metric derived from the aforementioned BLASTn analysis (Supplementary Table S6). For this analysis, we categorized all V/J genes into three groups: inWES (genes detected by WES), outWES-RNA (genes not detected by WES but detected by RNA-seq), and outWES-IR (genes not detected by WES but detected by IR-seq). Initially, we observed no significant differences among these three groups concerning HSP length or the percentage of HSP length relative to probe length (qcovhsp) (Fig. 3B). However, the HSP’s matching characteristics between the probes and the V genes showed highly significant differences. Specifically, in terms of the proportion of identical bases (pident) and the similarity score (bit score), the genes in the inWES group exhibited much larger values than those in the outWES-RNA and outWES-IR groups. Conversely, the inWES group showed a significantly smaller number of gap openings (gapopen) and bases in total gap openings (gaps) compared to the other two groups (Fig. 3C). Meanwhile, the J genes also displayed a similar trend to the V genes regarding the bit score differences among the groups (Fig. 3C).

We then examined V and J genes whose genomic coordinate regions did not overlap with any WES probe but were nevertheless detected in WES as part of the inWES group. These genes showed heterogeneous HSP similarity patterns (Supplementary Fig. S6). Several V genes still showed relatively high qcovhsp and/or large bit scores, while several J genes showed high pident but low qcovhsp. These results provide additional context for the small subset of V and J genes detected in WES despite lacking genomic-coordinate overlap with WES probes.

In summary, the analysis indicates a link between the nondetection of certain V/J genes in WES clonotype identification results and probe matching efficiency. The comparatively small number of clonotypes identifiable by the WES method is attributed to the fact that certain V and J genes lack probes with sufficiently high sequence matching accuracy.

### Validation of limited whole-exome sequencing clonotype detection using multiple independent paired datasets

To further validate the finding that WES yields a limited number of clones, we performed an external validation using an extensive collection of public datasets from various sources. This collection included human tissue samples from four different cancer types and one cardiac disease study, where each sample had paired WES and RNA-seq sequencing results (Supplementary Table S7). All analyses were performed across seven chains of TCR and BCR: TRA, TRB, TRD, TRG, IGH, IGK, and IGL. We then used the same pipeline to process both the WES and RNA-seq sequencing data, followed by a direct comparison of the receptor clonotype results derived from the two methods (Supplementary Table S8).

**Figure 4:**
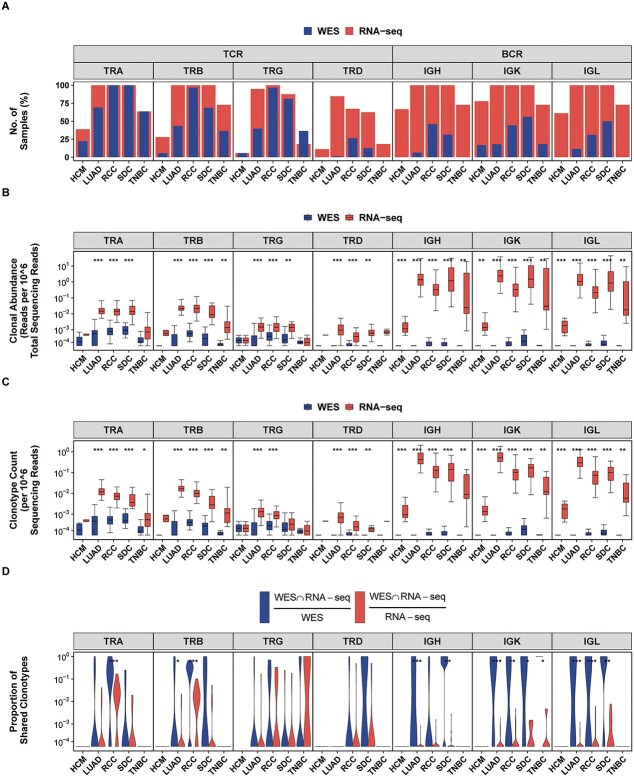
Comparative performance of WES and RNA-seq for clonotype identification in multiple validation datasets. (A) Percentage of samples with successful TCR/BCR clonotype identification across paired WES and RNA-seq datasets. (B) Box plots of clonal abundance (number of reads belonging to clonotypes per 10^6^ sequencing reads). (C) Box plots of clonotype count (number of identified clonotypes per 10^6^ sequencing reads). (D) Violin plots of clonotype overlap proportions between WES and RNA-seq. For each sample, overlap proportions were calculated as (WES∩RNA-seq)/WES for WES-derived clonotypes and (WES∩RNA-seq)/RNA-seq for RNA-seq-derived clonotypes, as indicated in the figure legend. Analyses were performed on five independent paired WES/RNA-seq validation datasets, labeled according to the disease context of their source studies: HCM (*n* = 18), LUAD (*n* = 78), RCC (*n* = 61), SDC (*n* = 16), and TNBC (*n* = 8). Detailed dataset information is provided in Supplementary Table S7. TRA, T-cell receptor alpha chain; TRB, T-cell receptor beta chain; TRD, T-cell receptor delta chain; TRG, T-cell receptor gamma chain; IGH, immunoglobulin heavy chain; IGK, immunoglobulin light-chain kappa; IGL, immunoglobulin light-chain lambda. Statistical comparisons: Wilcoxon signed-rank test; ^*^P < .05, ^**^P < .01, ^***^P < .001.

Consistent with the trends observed in the HCC datasets, the proportion of samples with successful clonotype identification using the validation datasets was generally lower for TRD than for other chains in both sequencing methods. Importantly, the detection proportion in WES results was lower than that in RNA-seq in most cases especially for BCR, with the exception of the RCC, SDC, and TNBC datasets for TRA (Fig. 4A). For clonal abundance and the number of identified clonotypes, the values obtained by WES were consistently lower than those obtained by RNA-seq in almost all cases. The only exceptions were the TRG chains in the HCM and TNBC datasets. This difference in quantification between the two methods was particularly pronounced in the BCR repertoire (Fig. 4B and C).

We next examined the shared clonotypes between the two methods for each sample. To quantify the overlap, we calculated the proportion of shared clonotypes by dividing the number of shared clonotypes by the number of clonotypes identified by each respective method. The results showed that the proportion of shared clonotypes in the TNBC and HCM datasets was extremely low, which could be attributed to relatively insufficient RNA-seq sequencing depth (Supplementary Table S7). Meanwhile, in other datasets, except for the TRD and TRG chains, the proportion of shared clonotypes in the WES results was generally higher than that in the RNA-seq results (Fig. 4D). This indicates that the WES method failed to detect a considerable proportion of clonotypes that could be identified using the RNA-seq method.

In summary, the comparative analysis of clonotype identification between WES and RNA-seq across multiple independent paired datasets showed a trend consistent with the findings in the HCC dataset, validating the underestimation of clonotype number and clonal abundance by the WES method.

## Discussion

This study systematically investigates the intrinsic technical constraints of WES for AIRR profiling, suggesting that incomplete probe design and low probe–target complementarity are factors linked to its suboptimal performance. Our genomic-coordinate and probe-sequence analyses show that WES probe sets provide incomplete coverage of the VDJ genes (Fig. 1C), which aligns with previous reports of substantial gaps in the effective coverage of standard WES platforms [15]. Specifically, approximately one-quarter of annotated VDJ genes were not directly targeted by probes in most platforms; conversely, some nontargeted loci—particularly nonfunctional paralogs—could nevertheless be captured when they share high sequence similarity with probes (Fig. 1D). By comparing BLAST-derived alignment metrics with the detection of V and J genes associated with productive CDR3 sequences, we found that those missed by WES exhibited significantly lower percent identity and bit scores, alongside higher gap penalties, compared to those successfully recovered (Fig. 3). These findings indicate that insufficient probe–gene complementarity largely explains the failure to detect many V/J genes in WES data. The successful identification of a CDR3 clonotype by WES is contingent upon the co-capture and complete assembly of its constituent V, D, and J genes. Consequently, genes with poor sequence complementarity to probes are less likely to be captured, thereby lowering the success rate of reconstructing the corresponding clonotype. Notably, D gene probes cover only a small fraction of the germline sequence across all platforms, which is substantially shorter than that observed for V and J genes (Fig. 1H). Since most germline D sequence is removed during VDJ recombination, the overlap would be even shorter in rearranged receptors, further limiting the likelihood of capturing D gene-derived clonotypes. This highlights an intrinsic limitation of WES for detecting D gene contributions to immune repertoires. It should also be noted that several metrics in Fig. 1, particularly the overlap-to-probe-length ratio and probe counts per gene, are strongly influenced by the intrinsic design strategies of different exome-capture platforms, including probe length, tiling density, and target-extension policies. This likely explains the distinct patterns observed between long V genes and short D/J genes in Fig. 1F and G, where longer probes frequently extend beyond D/J gene boundaries despite still providing substantial local coverage.

Previous efforts have attempted to mitigate these capture-related biases. Iyer *et al*. incorporated TCR-targeted probes into exome-capture protocols; however, this modification yielded only marginal increases in the number of recoverable TRA/TRB clonotypes. Clonotype counts scaled approximately linearly with sequencing depth and showed no evidence of saturation even at ~800× coverage (≈ 350 million reads); at that depth, whole-blood samples yielded only ~390 TRA and ~ 109 TRB clonotypes [16]. Similarly, the EXaCT-2 platform—an enhanced Agilent-based capture system—has been reported to achieve ≈ 98% nominal BCR gene coverage and to improve sensitivity for low-frequency clonotypes [17]. Levy *et al*. analyzed TCGA breast cancer exomes (using the Agilent SureSelect capture panel) and detected rearranged CDR3 reads in only 44% of tumors, with the majority of positive cases harboring fewer than 10 distinct clonotypes [18]. Collectively, these observations indicate that probe augmentation and capture refinements can modestly expand locus coverage and increase sensitivity to rare clones, but do not fully overcome the fundamental limits imposed by target locus complexity, transcript abundance, and the practical constraints of sequencing depth.

Beyond probe design and capture limitations, a more fundamental constraint stems from the nature of the source material. WES samples genomic DNA at fixed, low-copy resolution and therefore lacks the natural transcript abundance that RNA-seq exploits [19]. This absence of transcript-level multiplicity substantially reduces WES sensitivity to low-abundance receptor sequences. To provide a rough quantitative sense of this disparity, we performed a simple estimation of AIRR detection efficiency across the three methods using the HCC dataset. Clonal read counts and clonotype counts were normalized to per million sequencing reads to account for differences in sequencing depth across methods (as described in the Results). As expected, for TCR chains, the median normalized clonal read count and clonotype count from RNA-seq were ~6.7-fold and 5.4-fold higher than those from WES, respectively, while the corresponding values from IR-seq exceeded those of WES by several orders of magnitude. Notably, the median normalized clonal read count for TRD in WES was zero, indicating that TRD clonotypes were virtually undetectable by WES at the sequencing depths employed. For IGH, the disparity was even more pronounced: the median normalized clonal read count and clonotype count from RNA-seq were ~1839-fold and 1058-fold higher than those from WES, respectively, with IR-seq exceeding WES by an even greater margin. The notably larger RNA-seq/WES disparity observed for IGH compared to TCR chains may partly be attributable to the higher degree of somatic hypermutation in immunoglobulin genes, which could progressively reduce sequence similarity between rearranged BCR sequences and germline probe targets, although differences in transcript abundance and immune-cell composition may also contribute. This insensitivity to low-abundance sequences is particularly consequential for immune repertoires, whose clone-size distributions are typically heavy-tailed [20]. In such a distribution, a small number of highly expanded clones account for a large fraction of reads, whereas a long tail of low-frequency clones comprises most unique clonotypes but contributes little to total DNA copy number, rendering WES inherently insensitive to the majority of clones present at low abundance. Consequently, the absolute number of clonotypes recovered from WES data in our results was often orders of magnitude lower than that recovered by RNA-seq or targeted IR-seq (Figs 2 and 4), implying that WES may not reliably recapitulate the full richness or clonal architecture of complex immune repertoires.

As a consequence of these technical limitations, WES consistently underperformed RNA-seq in HCC datasets across multiple quantitative metrics: number of samples with detected clonotypes, total number of clonotypes recovered, clonal abundance, and repertoire similarity to the IR-seq gold standard (Fig. 2). The same pattern was consistently observed across several independent paired WES/RNA-seq datasets from diverse disease contexts (Fig. 4). These external validations indicate that limited clonotype recovery is a common limitation of WES data, regardless of disease contexts or laboratory protocols.

Nevertheless, our data and prior studies (see Supplementary Table S1) show that WES can recover some clonotypes of dominant immune responses. The partial overlap of clonotypes observed between WES and RNA-seq/IR-seq (Figs 2E and 4D) supports the interpretation that many clonotypes detected in WES data are genuine. That said, the limited sensitivity and severe bias of WES restrict its utility for rigorous, quantitative immune monitoring: exome-derived repertoires tend to be highly skewed toward a few expanded clones and miss the vast majority of low-frequency diversity. Consequently, WES may not be assumed to provide comprehensive or consistently quantitative profiles of immune-repertoire diversity.

Several limitations of the present analysis should be noted. In the field of AIRR research, the vast majority of studies perform clonotype identification at the gene level rather than the allele level. This reflects several practical constraints: allelic variants of VDJ genes often differ by only one or a few nucleotides, making them difficult to distinguish from sequencing errors; mainstream analysis tools, including MiXCR, report gene-level assignments by default; and existing reference databases remain incomplete with respect to interindividual allelic polymorphism. Consistent with common practice in the field, the present analysis was conducted entirely at the gene level. In this context, the ^*^00 allele notation used in MiXCR refers to a consensus sequence derived from all known alleles of a given gene, serving as a gene-level representative rather than any specific allelic variant. Since VDJ loci are among the most polymorphic regions in the human genome, the use of a single representative sequence inevitably fails to capture the full extent of interindividual sequence diversity and its potential impact on probe capture efficiency. This limitation is particularly relevant for genes in which functional and nonfunctional alleles coexist across individuals, as gene-level ORF+/ORF− classification cannot reflect such interindividual variation. It is worth noting that the suitability of different probe sets may therefore vary across individuals depending on their specific genotypes at these loci. Furthermore, for immunoglobulin genes, somatic hypermutation is expected to progressively reduce sequence similarity between rearranged receptors and germline probe sequences, meaning that WES capture efficiency for highly mutated BCR clonotypes may be lower than our analysis predicts. Whether such clonotypes are proportionally more detectable in RNA-seq than in WES data would be an interesting question for future investigation. Similarly, whether VDJ genes that differ from the reference probe sequences are less likely to be detected could be assessed through germline genotyping approaches, and represents a worthwhile direction for future analysis.

In summary, conventional WES is inherently limited for comprehensive characterization of AIRR because of two constraints: incomplete and uneven probe coverage across VDJ loci, and the low-copy nature of receptor loci in genomic DNA relative to the transcriptome. Although existing WES datasets can identify hyperexpanded clones, such observations may not be universally representative. Consequently, interpretations based on WES-derived repertoire signals should be framed cautiously. For mining clinical repositories aimed at quantitative immune monitoring, RNA-seq or IR-seq datasets remain the superior choice compared to WES.

## Materials and methods

### Genome position-based probe coverage analysis

The list of VDJ gene names was retrieved from the MiXCR internal library (repseqio v4.0), which is bundled within mixcr.jar (extractable as a standard ZIP archive) under the libraries/ directory, as distributed with MiXCR v4.6.0 (https://github.com/milaboratory/mixcr/releases/tag/v4.6.0). Repseqio v4.0 was used to ensure compatibility with MiXCR v4.6.0 and reproducibility of clonotype assignments. Corresponding gene annotation fields including chromosome, start and end coordinates, and functional status (ORF+/ORF−) were obtained using the BiomaRt R package (v2.60.1) referencing the Ensembl release GRCh38 v113, chosen to ensure consistency with commercial WES probe genomic coordinates. The BED files, which record the probes’ target region for six commercial exome-capture platforms, were downloaded from the respective vendors’ websites or the UCSC Table Browser [21] (Supplementary Table S2).

Probe overlapping analysis was performed by the GenomicRanges R package (v1.56.1). The `*GRanges*` objects were constructed for each VDJ gene and each probe of the six platforms based on their coordinate information. Probe–gene overlaps were identified by the function `*findOverlaps*`. To avoid double-counting when multiple probes overlap the same genomic segment, the function `*reduce`* was deployed to merge the overlaps and to compute the total overlap length for each gene (Supplementary Table S3).

To assess probe coverage at the biologically relevant ends of V and J genes, all V genes were aligned relative to their 3′ end and all J genes relative to their 5′ end, with position 0 defined as the respective recombination-proximal end in each case. For each relative position, the percentage of genes with coverage from at least one probe was calculated as the number of genes overlapping at least one probe at that position divided by the total number of genes in the respective gene type. Strand orientation was accounted for by converting genomic coordinates to relative positions based on each gene’s transcriptional direction.

### BLAST-based probe coverage analysis

Based on the probe coordinates provided in the BED files, the nucleotide sequences of probes were extracted from the human genome reference sequence (BSgenome.Hsapiens.UCSC.hg38) at corresponding positions using the Biostrings R package (v2.76.0).

The sequences of VDJ genes were also obtained from the MiXCR internal library (repseqio v4.0), utilizing only the ^*^00 alleles. Where nucleotide sequences were absent (e.g. IGHV3-53^*^00 and IGHV3-64D^*^00), the missing sequences were reconstructed following the tool’s protocol based on their mutation information.

The probe sequences were used as queries and aligned against a subject database constructed from the VDJ gene sequences, utilizing the BLASTn program from BLAST+ (v2.17.0). The HSP outputs were then parsed to extract the relevant similarity metrics.

### IR-seq and RNA-seq data generation of hepatocellular carcinoma samples

For IR-seq of HCC samples, a 5′ RACE strategy and two-round PCR were performed as previously described [22]. Briefly, cDNA synthesis was performed using 1.5 μg of total RNA isolated from 50 to 100 mg of HCC tissue. TCR α/β/γ/δ chains and BCR IgG/IgM/IgA heavy chains were amplified using universal forward primers and chain-specific reverse primers. PCR products were gel-extracted and purified using the QIAquick Gel Extraction kit (Qiagen, Germany). After ligation of Illumina adaptors and barcodes, high-throughput sequencing was performed on the Illumina HiSeq platform with a read length of 2 × 250 bp.

For RNA-seq of the same HCC samples, tissue RNA was extracted and rRNA was removed. The rRNA-depleted RNA was fragmented into 250–300 bp pieces, and cDNA synthesis was performed using fragmented RNA as template with random oligonucleotides. The cDNA was purified, end-repaired, ligated with sequencing adapters, and fragments of ~370–420 bp were purified using AMPure XP beads (Beckman Coulter, USA). PCR amplification was performed to obtain the final library, which was validated and sequenced on the Illumina HiSeq platform with a read length of 2 × 150 bp.

### Multi-omics sequencing dataset collection

The HCC dataset used in this study originated from a project we participated in [12]. The IR-seq sequencing data for this project have been deposited in the Genome Sequence Archive in the National Genomics Data Center, China (https://ngdc.cncb.ac.cn/gsa/) under the accession number HRA004388. Access to the paired WES and RNA-seq sequencing data requires application to the original project manager. For external validation, publicly available datasets were identified through the European Nucleotide Archive (ENA) (https://www.ebi.ac.uk/ena/browser/home) using the keywords “WES” and “RNA-seq.” We selected the datasets that provided clearly paired WES and RNA-seq sample information and possessed sufficient sample sizes for robust analysis. The BioProject accession numbers (National Center for Biotechnology Information, NCBI) for these selected datasets [23–27] are provided in Supplementary Table S7.

### TCR and BCR clonotype identification

TCR and BCR clonotype identification was performed using the MiXCR software (v4.6.0) [28]. The standard MiXCR workflow was executed, which comprises three main steps: sequence alignment to the reference germline genes, local assembly of CDR3 sequences, and output generation. The platform-specific command lines used for each sequencing type were as follows:

For IR-seq data:

mixcr analyze generic-amplicon --species hsa --rna --rigid-left-alignment-boundary --floating-right-alignment-boundary C ${input_R1_fastq} ${input_R2_fastq} ${output_dir}

For RNA-seq data:

mixcr analyze rna-seq --species hsa ${input_R1_fastq} ${input_R2_fastq} ${output_dir}

For WES data:

mixcr analyze exome-seq --species hsa ${input_R1_fastq} ${input_R2_fastq} ${output_dir}

In the output results, nonproductive CDR3 sequences (those that were out-of-frame or contained stop codons) were excluded. Only sequencing reads with productive CDR3 regions were retained to generate CDR3 amino acid sequence. A clonotype was defined as a unique CDR3 amino acid sequence within a sample, and the number of reads belonging to a specific clonotype was taken as its abundance.

### Statistical analysis

To enable cross-sample comparisons and mitigate variation in sequencing depth, both clonal abundance and clonotype count were normalized to the total number of sequencing reads for each sample, and were reported as values per million reads.

The MHSI to assess the consistency of the clonotype abundance profiles between pairwise methods was calculated as:


$$MHSI=\frac{2\sum_{i=1}^R{x}_i{y}_i}{\left({\lambda}_x+{\lambda}_y\right){N}_x{N}_y}$$

$$\mathrm{where},\ {N}_x={\sum_{i=1}^R}{x}_i,\ {N}_y={\sum_{i=1}^R}{y}_i,\ {\lambda}_x=\frac{\sum_{i=1}^R{x}_i^2}{N_x^2},{\lambda}_y=\frac{\sum_{i=1}^R{y}_i^2}{N_y^2}$$

Here, ${x}_i$ and ${y}_i$ represent the abundances of the *i-*th clonotype in methods X and Y, respectively, and $R$ is the number of clonotypes observed in both methods.

The evenness of the repertoire was evaluated using the NSDE calculated as:


$$NSDE=-{\sum_{i=1}^N}\frac{p_i\mathit{\ln}\ {p}_i}{\mathit{\ln}\ N}\ where,\ {p}_i=\frac{n_i}{\sum_{i=1}^N{n}_i}$$

Here, ${n}_i$ is the abundance of the *i-*th clonotype, and *N* is the total number of clonotypes (NSDE is equivalent to Pielou’s evenness index [29]).

Differences between two groups were evaluated using the Wilcoxon signed-rank test or the Wilcoxon rank sum test. Correlations between pairwise methods were assessed using Spearman’s rank correlation coefficient. All statistical tests were two-sided, with a *P*-value <.05 considered statistically significant.

**Key Points**

- Existing WES datasets have been repurposed for AIRR analysis, yet the methodological reliability of this application has not been systematically evaluated.
- Multi-omics benchmarking against IR-seq reveals that WES substantially underestimates clonotype richness and distorts clonal abundance compared with both RNA-seq and IR-seq.
- Incomplete and nonuniform capture of V, D, and J gene segments, together with insufficient probe–gene sequence complementarity, represents a major technical constraint underlying WES-based repertoire inference.
- These limitations are consistently observed across multiple independent public datasets, indicating that restricted clonotype recovery is an intrinsic property of conventional WES rather than a dataset-specific effect.
- Quantitative AIRR analysis should prioritize targeted IR-seq or RNA-seq, as repertoire signals derived from WES require cautious interpretation due to inherent limitations.

## Supplementary Material

Supplementary_material_bbag419

## Data Availability

The IR-seq sequencing data associated with this study have been deposited in the Genome Sequence Archive (GSA) at the National Genomics Data Center (NGDC), China (https://ngdc.cncb.ac.cn/gsa/) under the accession number HRA004388. Access to the paired WES and RNA-seq sequencing data from the HCC dataset [12] is available upon reasonable request through a formal application to the original project manager. Furthermore, the National Center for Biotechnology Information (NCBI) BioProject accession numbers for the external validation datasets are provided in Supplementary Table S7.

## Notes

Author description: The vast accumulation of whole-exome sequencing (WES) data in clinical repositories has led to numerous studies aiming to extract immune-repertoire information from these nontargeted datasets, yet their analytical reliability for this purpose remains poorly characterized. Using multi-omics benchmarking against IR-seq–derived clonotypes, we show that WES systematically underestimates repertoire richness and distorts clonal abundance when compared to both RNA-seq and IR-seq. These limitations are associated with the degree of sequence mismatch between commercial exome-capture probes and VDJ genes, which hinder efficient capture and consequently reduce clonotype recovery. We therefore emphasize the need for rigorous methodological caution in WES-based quantitative immune-monitoring applications.
